# Vitamin D Supplementation for Prevention of Type 2 Diabetes Mellitus: To D or Not to D?

**DOI:** 10.1210/clinem/dgaa594

**Published:** 2020-08-26

**Authors:** Anastassios G Pittas, Rolf Jorde, Tetsuya Kawahara, Bess Dawson-Hughes

**Affiliations:** 1 Division of Endocrinology, Diabetes and Metabolism, Tufts Medical Center, Boston, Massachusetts; 2 Institute of Clinical Medicine, UiT The Arctic University of Norway, Tromsø, Norway; 3 Division of Internal Medicine, Kokura Medical Association Health Testing Center, Kitakyushu, Japan

**Keywords:** vitamin D, diabetes, prediabetes, type 2 diabetes, prevention

## Abstract

**Context:**

Over the last decade, vitamin D has emerged as a risk determinant for type 2 diabetes and vitamin D supplementation has been hypothesized as a potential intervention to lower diabetes risk. Recently, several trials have reported on the effect of vitamin D supplementation on diabetes prevention in people with prediabetes.

**Evidence Acquisition:**

A comprehensive literature review was performed using PubMed, Embase, and ClinicalTrials.gov to identify: (1) recent meta-analyses of longitudinal observational studies that report on the association between blood 25-hydroxyvitamin D (25[OH]D) level and incident diabetes, and (2) clinical trials of adults with prediabetes that have reported on the effect of vitamin D supplementation on incident diabetes.

**Evidence Synthesis:**

Longitudinal observational studies report highly consistent associations between higher blood 25(OH)D levels and a lower risk of incident diabetes in diverse populations, including populations with prediabetes. Trials in persons with prediabetes show risk reduction in incident diabetes with vitamin D supplementation. In the 3 large trials that were specifically designed and conducted for the prevention of diabetes, vitamin D supplementation, when compared with placebo, reduced the risk of developing diabetes by 10% to 13% in persons with prediabetes not selected for vitamin D deficiency.

**Conclusions:**

Results from recent trials are congruent with a large body of evidence from observational studies indicating that vitamin D has a role in modulating diabetes risk. Participant-level meta-analysis of the 3 largest trials should provide a more refined estimate of risk reduction and identify patient populations that are likely to benefit the most from vitamin D supplementation.

Diabetes is one of the fastest growing health challenges of the 21^st^ century, as the number of adults living with diabetes has more than tripled over the past 20 years. It is estimated that 9.3% of adults aged 20–79 years worldwide (approximately 463 million) and 12% of US adults aged older than 18 years (approximately 30 million) are living with diabetes (1). More than 9/10 people with diabetes have type 2 diabetes. An estimated one-third of the adult US population (approximately 84 million) is at risk for type 2 diabetes (ie, have prediabetes), based on having a fasting glucose (FG) or hemoglobin A1c (HbA1c) level above the normal range but below the threshold for diabetes (2, 3). People with prediabetes progress to diabetes at a rate of about 5% to 10% per year (4). Persons at high risk for type 2 diabetes who are overweight or obese and who have an impaired glucose tolerance and elevated FG levels can slow the progression to diabetes with intensive lifestyle changes that lead to weight loss (5). However, sustaining lifestyle changes long-term is challenging, and there is always residual risk even after successful weight loss and maintenance. Therefore, simple, inexpensive, and sustainable approaches that can be applied at the public health level to complement lifestyle changes are needed to lower the risk of type 2 diabetes in persons with prediabetes.

Over the last decade, low blood 25-hydroxyvitamin D (25[OH]D) level has emerged as a risk factor for type 2 diabetes, and vitamin D supplementation has been hypothesized as a potential intervention to lower diabetes risk (6, 7). Observational studies strongly support an inverse association between blood 25(OH)D level and risk of developing type 2 diabetes in diverse cohorts of variable diabetes risk, especially in persons with prediabetes (8, 9). Results from short-term mechanistic studies offer a biologic plausibility to the hypothesis (10, 11). Recently, several trials testing the effect of vitamin D supplementation to reduce the rate of progression to diabetes in people with prediabetes have been completed (12–19).

We sought to evaluate and synthesize the available evidence to determine the role of vitamin D in the prevention of type 2 diabetes.

## Review Strategy and Literature Search

Although results from high-quality randomized controlled trials are typically considered to be the highest level of evidence to establish whether an intervention has an effect on the outcome of interest, the totality of evidence from different lines of research should be considered when establishing causality. In synthesizing the available evidence, we have used the Bradford Hill general guidelines for causality and have structured our review to address these guidelines in relation to vitamin D and the prevention of type 2 diabetes (Table 1) (20).

**Table 1.** Prerequisites for Effect (Causal Association) of Vitamin D for Prevention of Type 2 Diabetes.

• Plausible mechanism(s)
• Temporal relationship
• Strength of the association
• Dose response
• Consideration of alternative explanations
• Experimental evidence (ie, clinical trials)
• Challenges of vitamin D clinical trials
• Coherence/consistency among studies

A comprehensive literature review was performed using PubMed, Embase, and ClinicalTrials.gov to identify: (1) recent meta-analyses of longitudinal observational studies that report on the association between blood 25(OH)D level and incident diabetes, and (2) clinical trials of adults with prediabetes that have reported on the effect of vitamin D supplementation on incident diabetes.

### Overview of vitamin D physiology and plausible mechanistic links to the pathophysiology of type 2 diabetes

Vitamin D, obtained either from oral sources or cutaneous biosynthesis upon sun exposure, is hydroxylated first in the liver to 25(OH)D, and then in the kidneys to become the active form 1,25-dihydroxyvitamin D (1,25[OH]_2_D). These vitamin D metabolites are transported in the circulation bound primarily to vitamin D binding protein and only a small fraction circulates in the free form. The free 1,25(OH)_2_D form binds to the nuclear vitamin D receptor (VDR), which regulates hundreds of genes (21). Circulating 25(OH)D has a long half-life, can be readily measured, and correlates well with known vitamin D effects; therefore, it is used in clinical and research settings as a marker of vitamin D status.

The main effect of vitamin D is to increase the intestinal absorption of calcium. Severe vitamin D deficiency leads to rickets in children and osteomalacia in adults. However, due to the wide tissue distribution of the VDR and extrarenal activation of 25(OH)D to 1,25(OH)_2_D, it is believed that vitamin D has extraskeletal effects (22). Accordingly, low blood 25(OH)D levels have been associated with numerous diseases, including the risk of developing type 2 diabetes (23).

The hypothesis that vitamin D status may influence the risk of type 2 diabetes is biologically plausible, because both impaired pancreatic beta-cell function and insulin resistance have been reported with low blood 25(OH)D levels (10). Importantly, critical tissues in the physiology of glucose homeostasis, such as the beta cell, express 1-alpha-hydroxylase (CYP27B1) and can convert inactive vitamin D to its active metabolite (24). Furthermore, vitamin D deficiency in mice leads to reduced insulin secretion that can be restored by vitamin D supplementation (25). Systemic inflammation is another component in the pathophysiology of type 2 diabetes, and low blood 25(OH)D levels have been associated with high levels of inflammatory markers (26).

In humans, mechanistic studies show inconsistent results. Vitamin D supplementation for participants at high risk for or with newly diagnosed type 2 diabetes has shown an effect (27) as well as no effect (28) on insulin sensitivity and secretion. However, such studies are inconclusive because they are underpowered; have included populations with sufficient vitamin D status, with a low risk for diabetes or with established diabetes; have co-administered vitamin D with other interventions, which may confound the effect of vitamin D; or have followed participants for short periods of time (about 2–6 months), which are likely inadequate to affect the pathophysiology of type 2 diabetes.

Despite basic research studies providing some support for mechanisms in favor of vitamin D having an effect on the pathophysiology of type 2 diabetes, one needs to be careful of arguments in favor of biologic rationale, as the research history is filled with large trials that did not confirm a hypothesis that had a strong biological rationale from basic research.

### Temporal relationship, strength of association, and dose response

Evidence to support the prerequisites of the temporal relationship, strength of the association, and dose response (Table 1) comes from observational studies. Many cross-sectional studies have reported inverse associations between vitamin D status and glucose intolerance; however, cross-sectional studies are not informative and can only be considered hypothesis-generating, as the directionality of the association cannot be established.

Several observational, longitudinal studies conducted in diverse cohorts have reported consistent inverse associations between blood 25(OH)D levels and the risk of incident diabetes. Results have been summarized in recent meta-analyses with similar findings. Song et al combined data from 21 longitudinal cohorts (total of 76 220 participants; 4996 incident diabetes cases) and estimated a 38% risk reduction for incident diabetes in the highest versus the lowest category of blood 25(OH)D level (8). The association did not differ by sex, duration of follow-up, cohort sample size, 25(OH)D assay method, or diabetes diagnostic criteria. Afzal et al combined data from 16 longitudinal cohorts (total of 72 204 participants; 4877 incident diabetes cases) and reported that the bottom quartile of blood 25(OH)D level was associated with a 50% higher risk for incident diabetes compared with the top quartile (29). Ye et al included data from 22 longitudinal cohorts (89 698 noncases; 8492 diabetes cases) and reported that a 10-ng/mL lower 25(OH)D level was associated with a 22% higher risk of incident diabetes (30).

Notably, in the observational studies, the highest category of 25(OH)D level (conferring the lowest risk of diabetes) was in the 25 to 30 ng/mL range, and the lowest category (conferring the highest risk of diabetes) was in the 10 to 15 ng/mL range. In the meta-analysis by Song et al, a spline regression model showed that higher blood 25(OH)D levels were monotonically associated with a lower diabetes risk, without an apparent plateau (8). However, few observational studies have included enough participants with 25(OH)D levels higher than 30 ng/mL; therefore, it is not clear whether achieving and maintaining higher 25(OH)D levels are associated with an even lower risk of diabetes.

Observational studies using Mendelian randomization approaches, which offer the potential advantage that the reported genetic associations with phenotypes may overcome the challenges of confounding and reverse causation, have shown inconsistent associations between certain alleles relevant to vitamin D physiology and incident type 2 diabetes (30–34). In a Danish study of 96 423 adults, genetic variants associated with low blood 25(OH)D levels predicted incident type 2 diabetes (33). However, other studies in different cohorts have reported no associations between genetic variants that specifically affect blood 25(OH)D level and incident diabetes (30–32, 34). Mendelian randomization studies center upon certain assumptions that may not apply to vitamin D. Specifically, the tested alleles accounted for less than 5% of the variance in blood 25(OH)D level. Furthermore, Mendelian randomization studies did not predict the amounts of bioavailable or biologically active vitamin D and cannot distinguish between endogenous versus exogenous sources of vitamin D or long-term versus short-term exposure to vitamin D. In the study by Afzal et al, variation in the *DHCR7* gene, which is associated with lower vitamin D biosynthesis, predicted risk of type 2 diabetes, suggesting that sustained, long-term exposure to vitamin D may be important for the prevention of diabetes (33). Mendelian randomization studies may also be confounded by pleiotropic effects of genetic variants and are further limited by the assumption of a linear association between genetic variants, blood 25(OH)D level, and diabetes risk, which may not hold. Despite their theoretical appeal, Mendelian randomization studies can neither support nor exclude a causal relationship between vitamin D and type 2 diabetes.

Most longitudinal observational studies have included people with an average risk of type 2 diabetes at baseline. The inverse association between blood 25(OH)D level and incident diabetes may be more pronounced among persons who are already at a high risk for type 2 diabetes (9).

### Consideration of alternative explanations

Despite very promising and consistent data from observational studies, relying on observational data alone to establish causality is not sufficient, as evidenced by carefully examining the multiple determinants of the one’s vitamin D status, as reflected in one’s blood 25(OH)D level.

**Figure 1.**
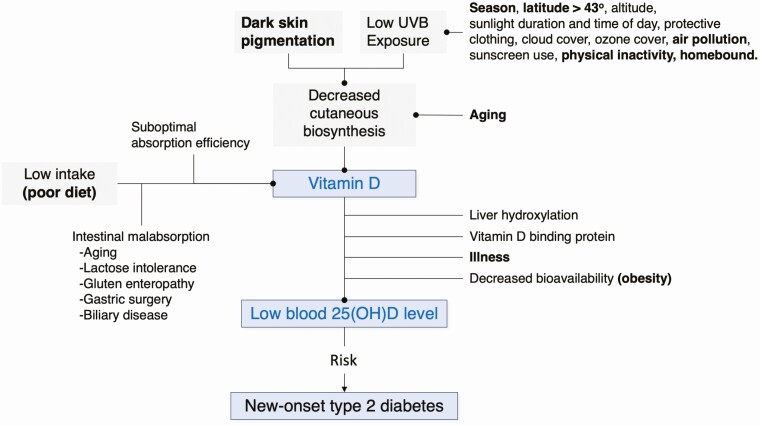
Factors (determinants) that contribute to low blood 25-hydroxyvitamin D (25[OH]D) concentration. Factors shown in bold are also associated with a risk of type 2 diabetes.

Vitamin D is obtained from oral sources or cutaneous biosynthesis and many factors influence each of these routes (Figure 1). The amount of vitamin D that reaches the circulation from oral sources is influenced by food selection, food fortification, supplement use, and absorption efficiency. For example, absorption is increased when supplemental vitamin D is co-ingested with a meal containing fat and it is decreased by medical conditions that produce intestinal malabsorption (35). Cutaneous biosynthesis declines with age and is reduced by higher levels of melanin in the skin (36). Season, latitude, altitude, time of day, ozone layer, and pollution also influence the degree of effective cutaneous biosynthesis. Individuals with a higher body weight have lower circulating vitamin D levels, probably reflecting the larger pool size over which vitamin D metabolites are distributed (37). Assuming that the supply of the precursor (vitamin D) is not rate-limiting, the blood 25(OH)D level reflects the balance between liver production, the genetically determined amount of vitamin D binding protein, and the metabolism of 25(OH)D to 1,24-dihydroxyvitamin D (1,24[OH]_2_D) or to 24,25-dihydroxyvitamin D (inactive metabolite).

Several factors that influence the blood 25(OH)D level also independently influence the risk of developing type 2 diabetes (Fig. 1). Chief among these are body weight and fatness, most frequently assessed by the body mass index (BMI). Specifically, a higher BMI is associated with both an increased risk of diabetes and a lower blood 25(OH)D level; hence, it is a potentially large source of confounding. Related to this, persons who are more physically active also tend to have a lower BMI and a lower risk of diabetes. When physical activity takes place out-of-doors, the increased cutaneous biosynthesis of vitamin D increases the circulating 25(OH)D level. Collectively, these factors are potential confounders in observational studies reporting on the association between blood 25(OH)D levels and the risk of developing type 2 diabetes.

Many other conditions, although not directly related to diabetes risk, affect 25(OH)D levels and may alter the reported association of 25(OH)D level with diabetes risk. For example, the association of 25(OH)D level with diabetes risk may not be linear, thus observational studies in populations that do not have a broad distribution of blood 25(OH)D levels may provide a misleading or incomplete picture of the true relationship between vitamin D status and diabetes risk.

### Experimental evidence (clinical trials)

There are 10 trials published between 2008 and 2019 that have reported on the effect of vitamin D supplementation on incident diabetes (12–19, 38, 39) (Table 2). Two large trials were designed and conducted for nondiabetes outcomes and reported data on incident diabetes in post hoc analyses (38, 39). These 2 trials enrolled patients who were at average risk for diabetes (ie, not prediabetes), the intervention included low-dose vitamin D (400 and 800 IU daily) co-administered with calcium, and diabetes was ascertained by participants’ self-report based on a diagnosis made outside of the study in routine clinical practice. Eight trials that have reported data on the effect of vitamin D supplementation and incident diabetes included persons with prediabetes (12–19). Five of these trials have major limitations, including a small sample size (109–205 participants) (12–15, 18), not being designed for incident diabetes as the primary outcome (12, 14, 15, 18), a short duration (≤1 year) (12, 14, 15, 18), and open-label study design (13, 15). Therefore, results from these 5 trials (12–15, 18) and the 2 large trials where incident diabetes was a post-hoc outcome (38, 39) are not informative in our understanding of the role of vitamin D supplementation for diabetes prevention in the clinical setting.

**Table 2.** Trials That Have Reported on Vitamin D Supplementation and New-onset Diabetes

First Author, Year of Publication (Country)	Participants	Baseline 25(OH)D Concentration	Interventions (Number of Participants)	Study Duration, Years	Study Quality (Reasoning)	Designed for Glycemic Outcomes?	Was Incident Diabetes the Primary Outcome (ie, Designed, Conducted, and Powered for Prevention of Diabetes)?
**Populations at average risk for diabetes**							
De Boer et al, 2008 (US)	Healthy, postmenopausal women (n = 33 951)	Not available	D_3_ 400 IU daily plus calcium carbonate 1000 mg daily (n = 16 999) vs placebo (n = 16 952)	Median of 7 years	Fair (post hoc analysis, self-report of diabetes, single gender)	No	No
Avenell et al, 2009 (UK)	Healthy, aged ≥70 years, history of fracture (n = 5292)	Not available	D_3_ 800 IU daily (n = 2649) vs placebo (n = 2643) [2x2 factorial design with calcium carbonate 1000 mg/daily]	Range of 2–5 years	Fair (post hoc analysis, self-report of diabetes)	No	No
**Populations with prediabetes**							
Davidson et al, 2013 (US)	Prediabetes and 25(OH)D < 30 ng/mL (n = 109)	22 ng/mL	D_3_ 88 865 IU weekly [~12 695 IU/d] (n = 56) vs placebo (n = 53)	Up to 1 year	Moderate (small sample size)	Yes	No
Dutta et al, 2014 (India)	Prediabetes and 25(OH)D < 25 ng/mL (n = 125)	17 ng/ml	D_3_ 60 000 IU weekly for 8 weeks, then monthly [~3000 daily over 12 months] (n = 68) vs no treatment (n = 57); all received calcium 1250 mg daily.	Mean of 2.3 years	Poor (small sample size, open label)	Yes	No
Barengolts et al, 2015 (US)	African American men with prediabetes and 25(OH)D 5–29 ng/mL and prevalent medical problems (n = 205)	14 ng/mL	D_2_ 50 000 IU weekly [~7142 UI daily] adjusted to achieve 25(OH)D 40–100 ng/mL (n = 103) vs placebo (n = 102). All participants received 400 IU of D_3_.	Up to 1 year	Fair (small sample size, single race and gender)	Yes	No
Kuchay et al, 2015 (North India)	Prediabetes (n = 137)	19 ng/mL	D_3_ 60 000 IU weekly for 4 weeks then monthly [~2000 IU daily over 12 months] (n = 69) vs no treatment (n = 68)	Up to 1 year	Poor (small sample size, open label)	Yes	No
Jorde et al, 2016 (Norway)	Prediabetes (n = 511)	24 ng/mL	D_3_ 20 000 IU weekly [~2857 IU daily] (n = 256) vs placebo (n = 255)	Up to 5 years	Good	Yes	Yes
Niroomand et al, 2018 (Iran)	Prediabetes and 25(OH)D < 30 ng/mL (n = 162)	13 ng/mL	D_3_ 50 000 IU weekly [~7143 IU daily] for 3 months then monthly (n = 81) vs placebo (n = 81)	Up to 6 months	Poor (small sample size, completers only analysis [50% of randomized])	Yes	No
Kawahara et al, 2018 (Japan)	Prediabetes (n = 1256)	Not available	Eldecalcitol 0.75 micrograms daily (n = 630) vs placebo (n = 626)	Up to 3 years	Good	Yes	Yes
Pittas et al, 2019 (US)	Prediabetes (n = 2423)	28 ng/mL	D_3_ 4000 IU daily (n = 1211) vs placebo (n = 1212)	Up to 4.5 years	Good	Yes	Yes

To convert the values of 25(OH)D to nmol/L, multiple by 2.496. The study quality for Kawahara et al was determined to be “good” based on the publication that describes the methods of the study. Study results have been published in abstract form only.

Abbreviations: 25(OH)D, 25-hydroxyvitamin D; US, United States.

Three double-blind, placebo-controlled, randomized trials have been designed and conducted specifically to test the hypothesis that vitamin D supplementation lowers the risk of diabetes among persons with prediabetes (16, 17, 19, 40, 41). Below, we describe these most relevant trials in detail.

#### The Tromsø study.

 The Tromsø study was a single-site trial that took place from March 2008 through March 2015 in Norway (16). The study randomly assigned 511 adults (mean age 62 years, mean BMI 30 kg/m^2^) who met at least 1 of 2 glycemic criteria for prediabetes (FG 108–125 mg/dL; glucose 2 hours after a 75-gram oral glucose load [2hG] 140–199 mg/dL) to treatment with 20 000 IU of vitamin D_3_ weekly (about 2857 IU per day) or placebo. The primary outcome was time-to-incident diabetes based on annual glycemic testing through FG, HbA1c, and 2hG. Mean baseline serum 25(OH)D level was 24 ng/mL (60.0 nmol/L) and 68% of participants had a level ≥20 ng/mL (49.1 nmol/L) (Table 3). During follow-up, mean serum 25(OH)D level in the vitamin D group rose to 44.1 ng/mL compared to 25.6 ng/mL in the placebo group. After a median follow-up of 2.5 years, 215 diabetes events had occurred: 103 in the vitamin D group and 112 in the placebo group (11.8 events vs 13.1 events per 100 person-years, respectively). In the intention-to-treat (ITT) analysis, the risk of diabetes was not significantly lower in the vitamin D group (hazard ratio 0.90; 95% confidence interval [CI]: 0.69–1.18) (16).

**Table 3.** Main Characteristics of the Key Randomized, Placebo-controlled Clinical Trials on Vitamin D Supplementation and Prevention of Diabetes Among Adults at Risk for Type 2 Diabetes (Prediabetes)

Study name, first author, year of publication (reference)	The Tromsø study, Jorde et al, 2016 (16)	The DPVD study, Kawahara et al, 2018 (17)	The D2d study, Pittas et al, 2019 (19)
Country (number of sites)	Norway (1 site)	Japan (3 sites)	United States (22 sites)
Year of trial completion	2015	2018	2018
Number of randomized participants	511	1256	2423
Prediabetes glycemic criteria for eligibility	IFG (FG 108–125 mg/dL) and/or IGT (2hG 140–199 mg/dL) and no criterion in the diabetes category	IGT (2hG 140–199 mg/dL) and no criterion in the diabetes category	Two or 3 glycemic criteria (IFG [FG 100–125 mg/dL], IGT [2hG 140–199 mg/dL], HbA1c 5.7–6.4%) and no criterion in the diabetes category
Serum 25(OH)D level, ng/mL	24	Not available	28
Participants with blood 25(OH)D level above 20 ng/mL, %	62	Not available	78
Intervention^a^	Cholecalciferol (vitamin D_3_), 20 000 IU weekly (~2857 IU daily) vs placebo	Eldecalcitol, 0.75 micrograms daily vs placebo	Cholecalciferol (vitamin D_3_), 4000 IU daily vs placebo
Vitamin D amount from supplements allowed outside of the study	≤400 IU/day	No amount was allowed	≤1000 IU/day
Definition of the primary outcome of new onset diabetes^b^	Any glycemic-positive criteria: FG ≥ 126 mg/dL, 2hG ≥ 200 mg/dL, HbA1c ≥ 6.5% (a positive HbA1c required confirmation)	HbA1c ≥ 6.5% and either: FG ≥ 126 mg/dL, 2hG ≥ 200 mg/dL, or casual glucose ≥ 200 mg/dL	Two or 3 glycemic-positive criteria: FG ≥ 126 mg/dL, 2hG ≥ 200 mg/dL, HbA1c ≥ 6.5%, or 1 criteria positive with confirmation
Expected incidence of diabetes in the placebo group, per 100 person-years	10.0	8.4	10.5
Expected relative risk reduction, vitamin D vs placebo, %	30	36	25
Median (range) duration of follow-up, years^c^	4 (0–5)	2.8 (not available)	2.5 (0–4.5)
Hazard ratio (95% CI) for incident diabetes, vitamin D vs placebo	0.90 (0.69–1.18)	0.87 (0.68–1.09)	0.88 (0.75–1.04)
Hazard ratio (95% CI) for incident diabetes among participants with starting serum 25(OH)D level < 12 ng/mL, vitamin D vs placebo	Data not available	Data not available	0.38 (0.18–0.80)

Abbreviations: 2hG, 2-hour glucose after a 75-gram oral glucose load; 25(OH)D, 25-hydroxyvitamin D; CI, confidence interval; FG, fasting glucose; HbA1c, hemoglobin A1c; IFG, impaired fasting glucose; IGT, impaired glucose tolerance.

^a^ Randomization ratio was 1:1 in all trials.

^b^ In the Tromsø study (N = 2 participants) and D2d study (N = 38 participants), diabetes was diagnosed outside of the study and confirmed by adjudication. In the DPVD study, no participant was diagnosed with diabetes outside of the study.

^c^ The Tromsø study followed participants for up to 5 years. The D2d study was designed as an event-driven trial and follow-up varied among participants. The DPVD study followed participants for up to 3 years.

#### Diabetes Prevention with Active Vitamin D study.

 The Diabetes Prevention with Active Vitamin D (DPVD) study was a 3-site trial that took place from June 2013 through August 2018 in Japan (17, 41). The study randomly assigned 1256 adults (mean age 61 years, mean BMI 24 kg/m^2^) who met the impaired glucose tolerance criterion for prediabetes (2hG 140–199 mg/dL) and had no diabetes (FG < 126 mg/dL and HbA1c < 6.5%) to treatment with 0.75 mg of eldecalcitol (an analog of the active form of vitamin D_3_) daily or placebo. The primary outcome was time-to-incident diabetes based on annual glycemic testing through FG, HbA1c and 2hG, and quarterly with FG and HbA1c. Information on mean baseline blood 25(OH)D level and proportion of participants with a level ≥20 ng/mL is not yet available. Based on results published in abstract form, after a median follow-up of 2.8 years, 121 diabetes events had occurred: 57 in the vitamin D group and 64 in the placebo group. In the ITT analysis, the risk of diabetes was not significantly lower in the vitamin D group (hazard ratio 0.87; 95% CI: 0.68–1.09) (17).

#### The Vitamin D and Type 2 Diabetes trial.

The Vitamin D and Type 2 Diabetes (D2d) study was a 22-site trial that took place from October 2013 through December 2018 in the United States (19, 40). The study randomly assigned 2423 adults (mean age 60 years, mean BMI 32 kg/m^2^) who met at least 2 of 3 glycemic criteria for prediabetes (FG 100–125 mg/dL; 2hG 140–199 mg/dL; HbA1c 5.7–6.4%) to treatment with 4000 IU of vitamin D_3_ daily or placebo. The primary outcome was time-to-incident diabetes based on annual glycemic testing through FG, HbA1c and 2hG, and semiannually with FG and HbA1c. The trial design was event-driven, with a target number of diabetes events of 508. Mean baseline serum 25(OH)D level was 28 ng/mL (69 nmol/L) and 78% of participants had a level ≥20 ng/mL (49 nmol/L) (Table 3). During follow-up, mean serum 25(OH)D level in the vitamin D group rose to 54 ng/mL compared with 29 ng/mL in the placebo group. After a median follow-up of 2.5 years, 616 diabetes events had occurred: 293 in the vitamin D group and 323 in the placebo group (9.4 events vs 10.7 events per 100 person-years, respectively). In the ITT analysis, the risk of diabetes was not significantly lower in the vitamin D group (hazard ratio 0.88; 95% CI: 0.75–1.04) (19).

In each of these 3 studies, protocol-specified adverse events of interest (hypercalcemia, hypercalciuria, and nephrolithiasis) were rare, and there were no significant differences between vitamin D and placebo.

### Challenges of vitamin D clinical trials

A clinical trial is often labeled as “positive” or “negative” based on whether the *P*-value for the statistical test for the primary outcome falls below or above (respectively) the traditional threshold of 0.05. This “dichotomania,” which is based on an arbitrary threshold, provides clarity for regulatory agencies when deciding whether to approve a pharmaceutical agent for clinical use; however, it is overly simplistic when trying to determine whether an intervention has a real and clinically meaningful effect (42). When the primary outcome in a clinical trial “fails,” there are several considerations that may clarify whether the intervention may still have clinical value (43). We modified a set of questions, described in the article by Pocock and Stone (43), for relevance to trials on vitamin D supplementation for the prevention of type 2 diabetes (Table 4). Below we address these questions in relation to the 3 major trials described above.

**Table 4.** Questions to Address When the Primary Outcome “Fails” in Trials of Vitamin D Supplementation for Diabetes Prevention

1. Is there some indication of potential benefit?
2. Was the trial underpowered?
3. Was the trial population appropriate?
4. Was the treatment regimen appropriate?
5. Was the primary outcome appropriate or accurately defined?
6. Was the intervention/follow-up duration appropriate?
7. Were there deficiencies in trial conduct (eg, under-recruitment, poor retention, poor adherence, use of rescue medication)?
8. Do subgroup findings elicit positive signals?
9. Can alternative analyses help?
10. Were there any safety issues with the intervention?

#### Is there some indication of potential benefit?

 When the primary outcome result is entirely neutral, concluding that the intervention has no effect is straightforward. Individually, each trial reported a result for the primary outcome of incident diabetes that did not reach the traditional 5% level of statistical significance; yet there is an indication of benefit from vitamin D, as all trials reported hazard ratios favoring the vitamin D treatment over placebo that were remarkably similar among the trials (0.87, 0.88, and 0.90).

#### Were the trials underpowered?

 Each trial was powered to detect a 25% to 36% relative risk reduction in incident diabetes with vitamin D supplementation compared with placebo (16, 40, 41). Based on the results, vitamin D supplementation appears to decrease diabetes risk among people with prediabetes not selected for vitamin D insufficiency by a smaller effect (~10–13%), but each trial was individually underpowered to test modest treatment effects. Of interest, in the Tromsø study and D2d study, the hypothesized relative risk reductions (30% and 25%, respectively) were within the reported 95% CI (0.69–1.18 and 0.75–1.04, respectively) (16, 19). Furthermore, as the size of the trial population increased, the reported 95% CI narrowed (Table 3). Therefore, it would be more appropriate to describe the findings of each individual trial as inconclusive rather than “negative.”

#### Were the trial populations appropriate?

 Selection of an appropriate trial population based on evidence from observational cohorts and mechanistic studies and good clinical judgment is essential. It has been suggested that vitamin D supplementation may be of benefit if started early in the natural history of type 2 diabetes. Despite its theoretical appeal, early supplementation presents multiple challenges. For example, diabetes progression is expected to be slow and vitamin D (or any intervention) is unlikely to have a detectable and clinically meaningful effect on diabetes risk if applied in people who are at an average risk for diabetes (38, 39). Furthermore, because the rate of progression from normal glucose tolerance to diabetes is highly dependent upon specific population characteristics, calculating study size and length of follow-up needed for such a clinical trial is complicated. For these reasons, all 3 trials appropriately enrolled people with prediabetes who are most likely to benefit from interventions to lower risk of progression to diabetes. Notably, all trials defined prediabetes by specific glycemic criteria (FG, HbA1c, 2hG), expanding their translational potential.

For ethical and practical reasons, the trials did not include blood 25(OH)D level as an eligibility criterion and did not measure blood 25(OH)D levels in real time. Because of frequent testing for vitamin D status in routine clinical settings and the widespread use of over-the-counter vitamin D supplements (44), trial participants were mostly vitamin D replete by current vitamin D recommendations (blood 25[OH]D level ≥ 20 ng/mL [50 nmol/L]) when the trials started and participants in the placebo group remained vitamin D replete during the follow-up (16, 19).

#### Were the treatment regimens appropriate?

 Determining the appropriate formulation and dose of vitamin D and timing of administration (eg, daily, weekly, monthly, yearly) in vitamin D trials can be challenging. The Tromsø study and D2d study administered vitamin D_3_ (cholecalciferol), the most commonly consumed vitamin D formulation, and thereby increased the studies’ translational potential. The DPVD study administered eldecalcitol, an analog of the active metabolite of vitamin D_3_ that does not require activation in the liver and kidney. Although infrequent dosing (eg, monthly, yearly) of vitamin D is convenient, it produces fluctuating blood 25(OH)D levels and is considered nonphysiologic (45, 46); therefore, daily or weekly dosing is preferred. The doses used (20 000 IU weekly [~2857 IU per day] in the Tromsø study, 0.75 mcg of eldecalcitol daily in the DPVD study, and 4000 IU daily in the D2d study) provide an appropriate balance of safety and efficacy in terms of obtaining an adequate difference in vitamin D status between the active and placebo groups.

All 3 trials appropriately compared vitamin D alone to placebo in a double-blind design. For practical and ethical reasons, all trials allowed participants to take outside-of-study vitamin D from all supplemental sources up to a certain amount and did not limit vitamin D intake from food sources. Many vitamin D trials have combined vitamin D with calcium or administered vitamin D in foods (eg, yogurt) and have used comparators other than placebo (13, 15, 38, 39). Such study designs are not informative regarding the role of vitamin D alone for diabetes prevention because they cannot isolate the effect of vitamin D from other components of the intervention.

#### Was the primary outcome appropriate or accurately defined?

 In all 3 trials, ascertainment for diabetes took place at regular intervals (every 3 or 6 months) by blood glucose testing using a single (central) laboratory and diabetes was appropriately defined based on strict, trial-specific glycemic criteria. Such an approach is robust and unbiased compared with other trials that relied on a self-reported diagnosis of diabetes outside of the study (38, 39), which is influenced by many uncontrolled factors that contribute random noise, potentially shifting the risk difference between vitamin D and placebo towards the null.

#### Was the duration of intervention and follow-up too short?

 Trial duration is an important consideration and one that requires careful weighing of competing factors. An adequate intervention period is essential given the long latency period for progression from prediabetes to diabetes and variable rate of progression (5–10% per year). Long-term trials encounter obligatory losses of participants due to a variety of unrelated intercurrent events. Secular changes in the perception of the value of the intervention may occur and lead to altered outside-of-study use of vitamin D, often at high doses. The adherence to study procedures may decline as participants experience study fatigue, leading to losing interest in continuing their participation. These occurrences reduce study power and increase the opportunity for postrandomization confounding.

The Tromsø study (mean 4 years, maximum 5 years), the DPVD study in Japan (mean 2.6 years, maximum 3 years), and the D2d study (mean 2.5 years, maximum 4.5 years) appear to have achieved a reasonable balance with regard to duration (16, 17, 19). In the D2d study, the Kaplan-Meir curves for incident diabetes in the vitamin D and placebo groups converged towards the end of the trial, indicating a low likelihood of a latent benefit from vitamin D supplementation.

#### Were there deficiencies in trial conduct?

 Based on full-text publications describing study results, the Tromsø study and D2d study were well conducted (16, 19). The design of the DPVD study is robust (41); however, results have been published in abstract form only (17), therefore the DPVD study conduct cannot be evaluated. All 3 trials met their recruitment goals. Retention in the Tromsø and D2d studies was excellent, with more than 99% of participants contributing follow-up data. Adherence to the trial regimen in the Tromsø and D2d studies was also excellent, with more than 85% of prescribed pills taken and fewer than 4% of trial participants taking out-of-study vitamin D supplements above the trial limit. In the D2d study, 1% of participants took diabetes or weight-loss medication before the diagnosis of diabetes was established. In the Tromsø and DPVD studies, none did.

#### Do subgroup findings elicit positive signals?

 Response to vitamin D depends on vitamin D status at baseline (47). Thus, people with higher baseline levels of blood 25(OH)D would benefit less from vitamin D supplementation than people with lower baseline levels (48). The high proportion of participants with adequate blood 25(OH)D levels at baseline may have prevented the detection of statistically significant differences between the vitamin D and placebo groups in the full trial cohorts. In all 3 trials, among participants with baseline blood 25(OH)D levels < 20 ng/mL, the risk of diabetes with vitamin D supplementation was reported to be lower compared with participants with levels ≥20 ng/mL, but the differences were not statistically significant. In the D2d study, the risk of diabetes was significantly lower in a small subgroup of participants with a baseline 25(OH)D level < 12 ng/mL (hazard ratio 0.38; 95% CI: 0.18–0.80; *p* for interaction = 0.023) (19). Results from subgroup analyses need to be interpreted cautiously partly because of the potential of a type I error (false positive) due to multiple analyses (49). However, given strong preexisting biologic plausibility, vitamin D supplementation may be more important in reducing diabetes risk among persons with prediabetes and low vitamin D status.

#### Can alternative analyses help?

 The ITT analyses are generally favored because of simplicity and because they alleviate concerns about confounding. However, although large scale trials are free of confounding when they start, biases may emerge during follow-up due to incomplete adherence to the trial intervention or use of rescue medications leading to postrandomization confounding, which may influence the estimate of treatment efficacy and study power (50, 51). The ITT analyses are agnostic to postrandomization confounding, including treatment discontinuation and concomitant therapies (eg, rescue medications such as high-dose vitamin D or metformin in trials for the prevention of diabetes with vitamin D) not allowed by the study protocol; hence, ITT analyses estimate the effect of treatment assignment, not the effect of treatment itself (51).

Differential adherence (for any reason) to the assigned intervention and concomitant exogenous use of vitamin D are common challenges in vitamin D trials given the widespread laboratory testing for blood 25(OH)D in the routine clinical setting and the availability of over-the-counter vitamin D supplements at high doses. These factors have the significant potential to influence the estimate of efficacy of vitamin D intervention for the prevention of diabetes in clinical trials. For example, in the D2d study, although overall adherence to the protocol was high and overall use of rescue medications was low, a different pattern among nonadherent participants emerged between the 2 groups. Specifically, during follow-up, more participants in the placebo group started diabetes or weight-loss medications, which would make the detection of diabetes less likely. Furthermore, more participants in the placebo group took personal vitamin D supplements above the trial limit, likely due to testing outside of the study. These differences may have shifted the relative risk reduction towards null in the ITT analysis. In a prespecified, per-protocol analysis that censored follow-up when a D2d participant started a diabetes or weight-loss medication, stopped study pills, or took out-of-study vitamin D from supplements that were above the study limit, the risk of diabetes was significantly lower in the vitamin D group (hazard ratio 0.84; 95% CI: 0.71–1.00) (19).

#### Were there any safety signals with the intervention?

 None of these trials reported a higher risk of adverse events with vitamin D supplementation versus placebo. In the Tromsø study, there were no significant differences in the protocol-specified adverse events of interest (hypercalcemia and kidney stones) or serious adverse events. The D2d study used cholecalciferol at 4000 IU/day, which is the tolerable upper intake level set by the National Academy of Medicine to avert potential toxicity. There were no significant group differences in the protocol-specified adverse events of interest (hypercalcemia, hypercalciuria, low glomerular filtration rate, and kidney stones) or serious adverse events. In abstract form, the DPVD study reported no serious adverse events with eldecalcitol, but the full determination of safety will await publication of the complete report.

## Coherence/Consistency Among Studies

Longitudinal observational studies show highly consistent associations between higher blood 25(OH)D levels and a lower risk of incident diabetes in diverse populations, including populations with prediabetes. Two meta-analyses from 2 different groups that combined aggregate data from trials on vitamin D for diabetes prevention were recently published. Zhang et al synthesized results from 8 trials (total of 4896 participants) in persons with prediabetes and reported a significant benefit of vitamin D supplementation for incident diabetes (risk ratio 0.89; 95% CI: 0.80–0.99) (52). The authors also reported that participants assigned to vitamin D supplementation were more likely to revert to euglycemia than the nonvitamin D group (risk ratio 1.48; 95%: CI 1.14–1.92). Barbarawi et al synthesized results from 9 trials (total of 43 559 participants). Two trials not designed for diabetes prevention (total of 39 243 participants) were in persons of average diabetes risk randomized to low-dose vitamin D (<1000 IU per day); 7 trials (total of 4316 participants) designed for diabetes prevention were in persons with prediabetes randomized to high-dose vitamin D (≥1000 IU per day). The authors reported a significant benefit of vitamin D supplementation for incident diabetes only after combining data from the diabetes prevention trials among persons with prediabetes who also received high-dose vitamin D (risk ratio 0.88; 95% CI: 0.79–0.99; p = 0.043) (53).

## Evidence Synthesis and Next Steps

Given the highly consistent results from the existing longitudinal observational studies, it is unlikely that new observational studies would modify the conclusion that vitamin D status is inversely associated with diabetes risk. A search of clinicaltrials.gov did not identify ongoing or planned randomized controlled trials specifically designed and being conducted to test the effect of vitamin D supplementation for the prevention of type 2 diabetes. Many large trials testing the effect of vitamin D supplementation on nondiabetes outcomes in populations at average risk for diabetes have been recently published or will be completed soon (54–58). We expect many of these trials to present secondary results on incident diabetes; however, these reports will require careful interpretation due to several limitations (eg, enrolled population at low/average risk, inadequately defined diabetes outcome). Therefore, the conclusions we draw on the role of vitamin D for the prevention of type 2 diabetes will depend on data we already have.

We also expect many of the completed trials to publish secondary results on the effect of vitamin D supplementation on micro- and macrovascular complications of diabetes. However, these trials are not powered for detecting an effect because the risk of developing micro- and macrovascular complications in these trial populations is very low.

Application of the Bradford Hill criteria to evaluate the totality of available evidence from longitudinal observational studies and clinical trials indicates a causal relation between vitamin D status and risk of type 2 diabetes. When combining data from the 3 large trials that were specifically designed and conducted to test vitamin D for diabetes prevention, Zhang et al reported a 12% reduction in diabetes risk with vitamin D supplementation (hazard ratio 0.88, 95% CI: 0.78–0.99) among participants with prediabetes not selected for vitamin D deficiency (52). Vitamin D supplementation may be more beneficial in adults with prediabetes and low vitamin D status, as suggested by the subgroup analysis in the D2d study that showed a 62% reduction (range 20–82%) in diabetes risk with vitamin D supplementation compared with placebo among participants with baseline serum 25(OH)D level < 12 ng/mL.

When evaluating the potential benefit of vitamin D supplementation, we should also not discount the benefit of the reversal of prediabetes to euglycemia. If vitamin D supplementation promotes regression to euglycemia, then more people will spend more time in the low-risk state away from prediabetes/diabetes. Therefore, the higher likelihood of reversal to euglycemia with vitamin D supplementation (~48% more likely, as stated by Zhang et al) may, by delaying time-to-onset of diabetes, represent an additional benefit to the 12% lower risk to progression to diabetes reported by the 2 meta-analyses of clinical trials (52, 53).

Although the summary results reported by Zhang et al and Barbarawi et al are concordant, meta-analyses that combine aggregate data from trials that vary in study design and quality should be interpreted cautiously (59). Individual participant data meta-analyses that combine data from high-quality clinical trials specifically designed and conducted to test the hypothesis are necessary to (1) estimate with precision the benefit of vitamin D supplementation on diabetes progression and regression to euglycemia, (2) assess heterogeneity of the treatment effect in order to define prediabetes subpopulations most likely to benefit most likely to benefit from vitamin D supplementation, and (3) evaluate safety using time-to-event analyses.

## Conclusion

Answers to clinically important questions are rarely dichotomous (“positive” or “negative”), and a recommendation of whether “to D or not to D” should be made based on the best available data from both observational studies and clinical trials. Results from trials are congruent with a large body of evidence from observational studies indicating that vitamin D has a role in modulating diabetes risk. We are awaiting the full publication of the DPVD study and results from individual participant data meta-analyses. Even if the risk reduction with vitamin D supplementation may appear relatively small, when applied in the expanding prediabetes population, it can have important public health implications.

## Data Availability

Data sharing is not applicable to this article, as no datasets were generated or analyzed during the current study.
